# Molecular Insights and Novel Therapies for Lymphoproliferative Disorders

**DOI:** 10.3390/ijms27115026

**Published:** 2026-06-02

**Authors:** Shucen Wan, Seema Naik

**Affiliations:** Penn State Milton S. Hershey Medical Center, 500 University Dr, Hershey, PA 17033, USA

**Keywords:** diffuse large B-cell lymphoma (DLBCL), follicular lymphoma (FL), mantle cell lymphoma (MCL), chimeric antigen receptor T cell therapy (CART), molecular mechanisms, hematological malignancies, targeted therapies

## Abstract

Hematological malignancies encompass a broad spectrum of relatively rare cancers with diverse biological and clinical characteristics that are capable of affecting individuals across all age groups, though certain subtypes show a predilection for specific age ranges. Advances in next-generation sequencing have greatly enhanced our understanding of the molecular and genetic basis of these diseases, while epigenetic, transcriptional, and proteomic analyses have further clarified their pathogenesis. These developments have shaped the classification and treatment of lymphoma. Updated classification frameworks which include the identification of clinically relevant molecular targets have opened the door to a number of targeted agents, each designed to exploit specific vulnerabilities within malignant cells, while stem cell transplantation continues to offer curative potential for eligible patients, with improving safety profiles over time. CAR-T-cell therapy has been extended to multiple blood cancer indications, achieving lasting remissions in patients with previously exhausted treatment options. Bispecific antibodies have further broadened the immunotherapy landscape by redirecting the body’s own T cells against tumor cells, offering a readily available alternative that overcomes many of the practical limitations associated with CAR-T-cell production. The ability to combine these strategies has fundamentally changed what is achievable in blood cancer treatment, with long-term remission now a realistic goal for many patients. This review seeks to outline the core molecular mechanisms underlying lymphoma and leukemia, evaluate currently approved treatment options, discuss significant ongoing clinical trials with practice-changing potential, and explore the prospect of chemotherapy-free approaches in carefully selected patient groups.

## 1. Introduction

Hematological malignancies encompass a broad spectrum of relatively rare cancers with diverse biological and clinical characteristics that are capable of affecting individuals across all age groups, though certain subtypes show a predilection for specific age ranges. Advances in next-generation sequencing have greatly enhanced our understanding of the molecular and genetic basis of these diseases, while epigenetic, transcriptional, and proteomic analyses have further clarified their pathogenesis [1,2]. The emergence of single-cell technologies and multiomics approaches [3,4], supported by robust bioinformatic tools, has provided deeper insight into disease initiation, progression, and treatment resistance. Collectively, these developments have not only improved diagnostic accuracy and enabled earlier disease detection, but have also transformed disease monitoring through liquid biopsy and circulating tumor DNA analysis, offering valuable prognostic and predictive information throughout the disease course [5,6]. These developments have shaped both how hematological malignancies are classified and how they are treated. The classification of hematological neoplasms has undergone significant transformation, driven by advances in molecular diagnostics. In 2022, two parallel classification systems emerged: the 5th edition WHO Classification (WHO-HAEM5) and the International Consensus Classification (ICC) [7,8,9,10]. Both systems updated prior frameworks through expert consensus, introducing new disease entities defined predominantly by genetic rather than morphological features. Lymphoid malignancies are stratified in both classifications by immunophenotypic lineage—B-cell, T-cell, and NK-cell—and further divided into precursor and mature neoplasms. Notably, the ICC places greater emphasis on genomic integration to facilitate precision-targeted therapy. Despite substantial concordance, the two systems retain differences in nomenclature and entity definitions.

**Figure 1:**
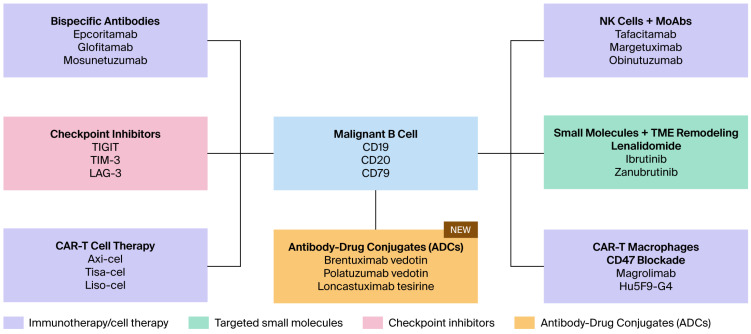
Novel therapies in lymphoma.

Understanding the molecular basis of these cancers has opened the door to a new era of treatment(shown in Figure 1 and Table 1). A growing number of targeted agents [11,12,13,14,15,16,17] are now available, each designed to exploit specific vulnerabilities within malignant cells. Immune-based strategies have equally transformed patient care, ranging from checkpoint blockade to engineered antibodies that recruit the immune system against tumor cells. Drugs that reverse abnormal epigenetic changes have shown particular benefit in patients with myeloid cancers [18], while stem cell transplantation continues to offer curative potential for eligible patients, with improving safety profiles over time. CAR-T-cell therapy [19], initially approved in 2017 for relapsed or refractory ALL, has since been extended to multiple blood cancer indications, achieving lasting remissions in patients with previously exhausted treatment options. Bispecific antibodies have further broadened the immunotherapy landscape by redirecting the body’s own T cells against tumor cells [20,21], offering a readily available alternative that overcomes many of the practical limitations associated with CAR-T-cell production. While immunotherapy is being broadly developed across various hematologic malignancies, including Hodgkin lymphoma, multiple myeloma, ALL, AML, and CLL, this review focuses specifically on immunotherapy developments in the NHL subtypes DLBCL, FL, MCL, and PTLD, where the most significant and rapidly evolving advances have been observed. The ability to combine these strategies has fundamentally changed what is achievable in blood cancer treatment, with long-term remission now a realistic goal for many patients.

Epigenetic disruption has emerged as a common thread across hematological malignancies. Abnormal DNA methylation, altered histone marks, defective chromatin remodeling, and dysregulated non-coding RNA networks collectively contribute to silencing tumor suppressor genes, activating oncogenic pathways, and maintaining cancer stem cell populations [22]. The targeting of these epigenetic vulnerabilities using agents such as DNA methyltransferase inhibitors, histone deacetylase inhibitors, *EZH2* inhibitors, and BET bromodomain inhibitors is increasingly being incorporated into combination treatment regimens [23].

This review seeks to outline the core molecular mechanisms underlying major hematological malignancies, evaluate currently approved treatment options(shown in Table 2), discuss significant ongoing clinical trials with practice-changing potential, and explore the prospect of chemotherapy-free approaches in carefully selected patient groups.

## 2. Molecular Mechanisms of Hematological Malignancies

### 2.1. Oncogenic Driver Mutations and Chromosomal Translocations

Among the initiating events in blood cancers, chromosomal translocations stand out as the most frequently encountered [24], giving rise to fusion proteins that interfere with normal transcriptional regulation, intracellular signaling, or cell cycle progression. The best-characterized example is the Philadelphia chromosome, resulting from a reciprocal translocation between chromosomes 9 and 22, which produces the *BCR-ABL1* fusion protein and drives uncontrolled cellular proliferation through persistent kinase activity [25]—a hallmark of CML and a subset of B-ALL cases. MCL is defined by the characteristic t(11;14)(q13;q32) translocation, which drives aberrant overexpression of *cyclin D1 (CCND1)* [26], thereby disrupting normal cell cycle regulation and contributing to malignant transformation. Additionally, in follicular lymphoma, BCL-2 upregulation renders malignant cells resistant to programmed cell death [27].

Beyond structural rearrangements, point mutations in critical driver genes play an equally important role in shaping disease behavior and informing treatment decisions. In AML, mutations affecting *FLT3*, *NPM1*, *IDH1/2*, *DNMT3A*, *TET2*, and *TP53* define clinically and biologically distinct patient subgroups, a complexity now formally recognized in current classification and risk stratification systems [28]. In CLL and MCL, the disruption of *TP53* [29,30]—whether through mutation or chromosomal deletion—carries the greatest adverse prognostic weight, underpinning resistance to chemotherapy and, in some MCL cases, BTK inhibition as well. Large-scale sequencing studies have significantly expanded the known mutational landscape of B-cell lymphomas. Whole-genome and whole-exome sequencing have uncovered 44 recurrently mutated genes and 11 somatic copy number variations implicated in CLL [31], including key genes such as *NOTCH1*, *TP53*, *ATM*, *SF3B1*, and *BIRC3*, among others [32,33]. These genetic alterations cluster into several critical pathways—*BCR* and toll-like receptor signaling, DNA damage response, *NOTCH1* signaling, apoptosis, *NF-κB* signaling, and RNA splicing—each playing a distinct role in CLL development and progression. Clinical studies have confirmed the prognostic relevance of these mutations, with *NOTCH1*, *SF3B1*, *BIRC3*, and *TP53* in particular linked to unmutated IGHV status and poorer patient outcomes [34], as reflected in shorter time to first treatment, reduced progression-free survival, and diminished overall survival.

### 2.2. Epigenetic Dysregulation

Epigenetic dysregulation has emerged as a central hallmark of hematological malignancies, manifesting principally through three interconnected mechanisms: aberrant DNA methylation patterning, disruption of histone modification networks, and dysregulated non-coding RNA-mediated transcriptional control [22]. In myeloid neoplasms, including AML [35] and MDS, unsupervised analysis of DNA methylation across AML samples revealed distinct mutation-specific patterns, with *IDH1/IDH2* mutations driving widespread methylation gains, while MLL fusions and co-occurring *NPM1*, *DNMT3A*, and *FLT3* mutations were associated with extensive methylation loss, relative to normal CD34+ CD38− cells. Across 160,519 CpG loci—42% of all sites tested—significant methylation changes were identified, with gains predominating (67%) over losses (33%) and approximately 71% of alterations occurring in coding regions, underscoring the broad epigenetic disruption characterizing AML [36].

In lymphoid malignancies, recurrent loss- or gain-of-function mutations targeting histone-modifying enzymes—notably *KMT2D*, *CREBBP*, *EP300*, *EZH2*, and *ARID1A*—disrupt the epigenetic programs that govern normal germinal center biology, thereby creating conditions permissive for lymphomagenesis. Among these, *EZH2* gain-of-function mutations, identified in approximately one fifth of diffuse large B-cell lymphoma (DLBCL) [37] and follicular lymphoma cases [38], pathologically amplify H3K27 trimethylation at key regulatory loci, suppressing differentiation and facilitating immune evasion. In contrast, loss-of-function mutations in *CREBBP* [39,40] compromise the transcriptional activation of MHC class II [41] and related immunogenic gene programs, a mechanism now increasingly implicated in resistance to immunotherapy-based treatment strategies.

### 2.3. BCR and Kinase Signaling Pathway Activation

BCR signaling represents one of the most therapeutically relevant survival pathways across B-cell malignancies. Activation of this pathway—whether through tonic baseline signaling or antigen-mediated stimulation—engages a cascade of downstream kinases including *SYK*, *PI3K*, and *BTK* which collectively converge on *NF-κB*, *MAPK*, and *AKT/mTOR* effector pathways to drive cellular proliferation, survival, and localization within supportive bone marrow and lymph node niches. In both CLL and MCL, the dependency on chronic active *BCR* signaling has been unequivocally validated by the transformative clinical efficacy of BTK inhibitors, which remain among the most impactful targeted therapies introduced into hematological oncology [42,43,44]. BTK inhibitors also show subtype-selective activity in DLBCL—meaningful benefit is restricted to ABC/MCD/N1 subtypes driven by chronic active *BCR* and *NF-κB* signaling. GCB-DLBCL does not respond [45,46]. The randomized, double-blind phase 3 ESCALADE trial (NCT04529772) and BELIEVE-01 trial (NCT05234684) both evaluate BTK inhibitors—Calquence^®^ (acalabrutinib) and orelabrutinib, respectively—in combination with R-CHOP versus placebo plus R-CHOP in previously untreated DLBCL patients, with expected completion dates of February 2027 and December 2025. In FL, early data with BTKi + bispecific combinations (zanubrutinib + mosunetuzumab, Pirtobrutinib and Mosunetuzumab) are very promising, with thorough responses, but data are still maturing, as evaluated in the phase II clinical trial (NCT05389293 and NCT06948786). These promising two-drug results have prompted investigators to ask whether adding a third targeted agent could further improve the depth and durability of response, forming the rationale for triplet combination strategies such as pirtobrutinib plus mosunetuzumab in the PROMOTE-FL trial [47].

Constitutively activating FLT3-ITD mutations, which occur in approximately one quarter to one third of AML patients, represent a dominant oncogenic driver through aberrant activation of *STAT5*, *PI3K*, and *MAPK* signaling cascades [48]. Similarly, the *JAK2V617F* point mutation—present in the vast majority of polycythemia vera [49] cases and a substantial proportion of essential thrombocythemia and myelofibrosis patients [50]—drives pathological *JAK-STAT* pathway activation, giving rise to the characteristic proliferative phenotype of myeloproliferative neoplasms.

### 2.4. Tumor Microenvironment and Immune Evasion

Hematological malignancies arise within a highly intricate tumor microenvironment (TME) composed of cancerous cells, stromal components, immune cells, and signaling molecules that work in concert to evade immune surveillance and drive resistance to treatment [51]. In CLL, the lymph node niche sustains malignant cell survival through physical interactions with nurse-like cells, regulatory T cells, and mesenchymal stromal cells. At the same time, this environment actively dampens anti-tumor immunity via CD200 expression [52], upregulation of PD-L1, and the secretion of immunosuppressive mediators such as IL-10 and TGF-beta [53].

Within DLBCL, tumor-associated macrophages (TAMs)—pro-tumorigenic (M2-like) [54,55] CD163+ TAMs—are associated with poorer clinical outcomes through multiple mechanisms. Beyond their well-established immunosuppressive role, these TAMs actively support tumor angiogenesis [56,57]; elevated CD163+ TAM density correlates with higher VEGF-A expression and increased intratumoral microvascular density. Separately, DLBCL tumor cells exploit the CD47/SIRPα axis as an innate immune checkpoint [58,59,60], overexpressing the “don’t eat me” signal CD47, which engages SIRPα on TAMs and inhibits macrophage-mediated phagocytosis. In patient-derived DLBCL biopsies, high SIRPα expression on TAMs correlates with impaired antibody-dependent cellular phagocytosis (ADCP), making this pathway a rational therapeutic target [61]. Across lymphoma subtypes, myeloid-derived suppressor cells (MDSCs), regulatory T cells (Tregs), and activation of the PD-1/PD-L1 checkpoint axis collectively reinforce an immunosuppressive milieu [62,63].

### 2.5. Clonal Evolution and Therapeutic Resistance

The clonal evolution of malignant cells—acting on pre-existing or treatment-induced subclones—constitutes the principal driver of therapeutic resistance across hematological malignancies. In CLL patients receiving BTK inhibitors, point mutations in BTK [64] (most notably C481S) or PLCG2 emerge in a substantial subset of individuals relapsing after ibrutinib therapy; these alterations shift binding kinetics to a non-covalent mode that renders the drug ineffective [65].

Complementing these insights, measurable residual disease (MRD) monitoring—harnessing ultra-sensitive next-generation flow cytometry or sequencing-based assays—has established itself as a vital instrument for longitudinally tracking clonal dynamics [66,67,68,69], capturing not only residual disease burden but also the real-time emergence of therapy-resistant subclones through clonal evolution and informing adaptive treatment decisions such as therapy de-escalation or intensification. Achieving MRD negativity at sensitivity thresholds of 10^−5^ or beyond has now been adopted as a validated surrogate endpoint across landmark clinical trials in CLL, AML, and ALL [70]. Shown in Table 3.

## 3. Diffuse Large B-Cell Lymphoma (DLBCL)

While R-CHOP cures approximately 60–70% of DLBCL patients, a significant subset relapse; develop refractory disease; or cannot tolerate treatment due to age, frailty, or comorbidities. High IPI scores and double/triple-hit lymphoma further limit outcomes, underscoring a critical unmet need. These gaps have spurred immunological approaches targeting key B-cell antigens—CD19, CD20, and CD79b—as well as the tumor microenvironment.

DLBCL, the most common lymphoid malignancy in adults, is a clinically and genetically heterogeneous disease further classified into transcriptionally defined activated B cell (ABC) and germinal center B cell (GCB) [71]. Currently, DLBCL that is not otherwise specified (NOS) must be distinguished from high-grade B-cell lymphomas, NOS, and from high-grade “double-hit” and/or “triple-hit” lymphomas. The latter are associated with dismal outcome and poor response to immunochemotherapy and transplantation. Of note, a subset of DLBCL-NOS referred to as double expressor DLBCL (DEL) [72,73,74,75] is defined by the co-expression of *MYC* (≥40%) and BCL2 (≥50%) on immunohistochemistry, distinct from double-hit lymphoma, which requires FISH-confirmed gene rearrangements. Double-expressor DLBCL-NOS demonstrates inferior outcome compared to those lacking *MYC/BCL2* overexpression, but is prognostically more favorable than “double-hit”/”triple-hit” lymphomas.

The advent of next-generation sequencing (NGS) has facilitated the development of prognostic classification systems for DLBCL based on somatic mutational profiles. In a landmark study, Chapuy et al. [76] performed an integrative genomic analysis of 304 primary DLBCL samples, evaluating 158 recurrent genetic alterations, including mutations, copy number changes, and structural variants. Their clustering approach identified five distinct molecular subtypes (C1–C5), as well as an additional group lacking defining genetic alterations. Using the GenClass algorithm, Schmitz and colleagues at the National Institutes of Health analyzed 574 DLBCL cases, identifying four genetically defined subtypes—MCD, N1, BN2, and EZB—based on patterns of genetic alterations [77]. Subsequent expansion of this approach in a larger cohort of over 1200 DLBCL samples led to the identification of two additional subtypes, A53 and ST2, further refining the molecular classification of this heterogeneous disease [78].

Within the activated B-cell-like (ABC) subtype of DLBCL, the N1 genetic subgroup is associated with the least favorable clinical outcomes. This specific category is defined by a distinct mutational profile, most notably involving alterations in the NOTCH1, ID3, and BCOR genes. Similar to the N1 category, the MCD [77] subtype is associated with poor clinical outcomes and shares significant overlap with Cluster C5 from the Chapuy classification [76]. Characteristically found in extranodal sites, MCD cases are defined by the co-occurrence of *MYD88* (p.L265P) and *CD79B* mutations. These genetic features, along with other concurrent alterations, synergistically promote *NF-κB* signaling [79], accelerate cell growth, inhibit programmed cell death, and allow the lymphoma to bypass immune detection.

Characterized by a “marginal zone-like” genetic signature, the BN2/Cluster C1 subtype is associated with a prognosis ranging from intermediate to favorable. This subgroup is defined by *BCL6* translocations, *NOTCH2* mutations, and various alterations that modulate the B-cell receptor (*BCR*) and *NF-κB* signaling pathways. Although it is primarily identified within the ABC-like cell-of-origin group, it is also notable for containing a significant number of DLBCL cases that are otherwise considered “unclassifiable” by traditional gene expression profiling.

Conversely, the *EZB*/Cluster C3 and ST2/Cluster C4 subtypes are restricted to the GCB-like phenotype. EZB is driven by *BCL2* translocations and mutations in epigenetic regulators and PI3K signaling. ST2 also involves epigenetic and PI3K alterations but adds *JAK/STAT* pathway mutations, mirroring the genetic landscape of NLPHL and THRLBCL. Clinically, the ST2/C4 group is associated with a significantly better prognosis than the EZB/C3 group.

The genetic characteristics of these newly defined subsets, including their mutational signatures and temporal sequence of alterations, offer novel insights into DLBCL pathogenesis. These coordinated genetic profiles predict clinical outcomes independently of the International Prognostic Index and suggest potential targeted combination therapeutic approaches. This molecular classification framework establishes a foundation for clinically actionable DLBCL stratification.

Emerging clinical trial evidence demonstrates the therapeutic implications of genotype-directed treatment strategies. Wilson et al. showed that adding ibrutinib to standard R-CHOP therapy achieved 100% three-year event-free survival in patients under 60 years with MCD and N1 subtypes [80]. Similarly, patients harboring unfavorable mutations in *PIM1*, *SPEN*, *or MYD88* (corresponding to BN2/Cluster C1 or MCD/Cluster C5 groups) or signatures involving *NF-κB*, *IRF4*, and *JAK-STAT* pathways (spanning multiple subtypes including BN2/Cluster C1 and ST2/Cluster C4) demonstrated improved outcomes when lenalidomide was added to R-CHOP as an immunomodulatory agent [81]. Additionally, patients with DLBCL characterized by distinct CD8+ T-cell signatures benefited from bortezomib combined with R-CHOP [82]. Conversely, the addition of venetoclax, a BCL2 inhibitor, to R-CHOP failed to improve outcomes in DLBCL patients with BCL2 overexpression (EZB/Cluster C3 or MCD/Cluster C5), underscoring the complex nature of DLBCL pathogenesis and the importance of precise molecular targeting strategies [83].

While NGS-based molecular subclassification—encompassing subtypes such as MCD, EZB, BN2, and N1—has profoundly advanced our biological understanding of DLBCL heterogeneity, its translation into routine treatment decisions remains an active area of development rather than established practice. Recent trials have begun implementing genetic subgrouping to optimize frontline DLBCL therapy. The phase II GUIDANCE-01 trial (*n* = 128) [84] randomized patients to R-CHOP versus R-CHOP plus a subtype-matched agent selected by a 20-gene LymphGen-like algorithm—ibrutinib for MCD/BN2-like, tucidinostat for EZB, decitabine for TP53-mutated, and lenalidomide for N1/NOS subtypes—demonstrating meaningful improvements in CRR, two-year PFS, and OS, though limited by its unvalidated algorithm, small size, open-label design, and single-center conduct [85]. The phase III GUIDANCE-02 trial (NCT05351346, *n* = 1100; 58 sites; 1043 patients randomized as of March 2025) addresses these shortcomings using the validated 38-gene LymphPlex algorithm [86] to assign orelabrutinib, lenalidomide, or decitabine to R-CHOP by subtype, with PFS as the primary endpoint.

## 4. Follicular Lymphoma (FL)

The treatment of follicular lymphoma (FL) has evolved due to the rapid expansion of immunotherapy options, with multiple agents now approved across different lines of therapy. Rituximab in combination as part of chemoimmunotherapy for frontline treatment followed by Rituximab maintenance continues to serve as the standard backbone of care.

Recently, Rituximab lenalidomide (R^2^) has become another attractive chemotherapy-free option. In another single-center phase 2 trial of ninety patients with previously untreated high-tumor-burden FL, the combination of obinutuzumab and lenalidomide demonstrated robust and durable efficacy. The primary endpoint of 2-year PFS was 93.3% (95% CI, 88.2–98.6), with median PFS not reached after a median follow-up of 70.7 months. The complete response rate at 30 months was 89.7% (95% CI, 81.3–95.2). Regarding tolerability, grade 3 or higher toxicities included neutropenia (18.9%), maculopapular rash (11.1%), and pneumonia (6.7%), overall reflecting a manageable safety profile [87].

The management of relapsed/refractory (R/R) follicular lymphoma has evolved considerably with the emergence of chemotherapy-free targeted strategies, demonstrating meaningful efficacy across multiple lines of therapy. The combination of rituximab and lenalidomide (R^2^) established a compelling benchmark in the randomized AUGMENT(NCT01938001) trial [88], achieving a median progression-free survival of 39.4 months versus 14.1 months with rituximab monotherapy, while substituting obinutuzumab in the GALEN study [89] yielded a numerically superior ORR of 79% and CR rate of 38%. In the BTK inhibitor space, zanubrutinib plus obinutuzumab demonstrated superiority over obinutuzumab alone in the ROSEWOOD trial (NCT03332017. ORR 68.3%; median PFS 27.4 months) [90], and the EZH2 inhibitor tazemetostat offered a molecularly informed option with preferential activity in EZH2-mutated patients (ORR 69%, CR 13%) [91]. Bispecific T-cell engager antibodies—including mosunetuzumab [92], epcoritamab [93], glofitamab [94,95], and odronextamab [96]—have yielded ORR 70–90% and CR rates of 50–75% even in heavily pretreated populations, while CAR-T-cell therapies [97,98,99] have produced thorough and durable remissions exceeding 86% ORR, albeit at the cost of significant toxicities including cytokine release syndrome, ICANS, and cytopenias. Collectively, while these advances represent a paradigm shift in the R/R FL landscape, the absence of head-to-head comparative data underscores the critical need for prospective trials to inform optimal treatment sequencing and patient selection.

The correlation between mutations affecting DNA damage response pathways *(TP53 and CDKN2A)* and transformation risk aligns with established understanding, as these mechanisms are well-characterized drivers of aggressive lymphoma development and broader cancer progression. Moreover, copy number alterations at TP53 and CDKN2A loci have been associated with poor outcomes in follicular lymphoma [100]. The modest associations observed between *GNA13* and *BTK* mutations with reduced time-to-transformation [101,102] and the *IRF8* mutations with extended time-to-transformation [103] suggest the involvement of pathways related to germinal center biology. Notably, *GNA13* loss has been identified as a gene with increased mutational frequency in transformed follicular lymphoma [104,105]. Despite our substantial cohort size, we found insufficient evidence to support associations between specific mutational clusters and transformation events. Although recurrent mutational patterns are identifiable in both follicular lymphoma and DLBCL, their prognostic utility in follicular lymphoma remains limited. This limitation reflects the heterogeneous management approaches and indolent clinical course characteristic of follicular lymphoma. Nevertheless, understanding the underlying mechanistic differences between these mutational patterns may reveal opportunities for developing molecular subtype-directed therapeutic strategies in future clinical applications.

Recently, CD20 × CD3 bispecific antibodies mosunetuzumab, epcoritamab, and odronextamab have been FDA-approved for third-line or later follicular lymphoma. In phase II trials, all three demonstrated overall response rates of ~80% and complete response rates of 60–73%, most of which were still maintained at two years. Common toxicities across agents include cytokine release syndrome occurring in half of patients, with rare grade 3 CRS. While efficacy and safety profiles are broadly similar across agents, key differences remain in treatment duration, route of administration, and corticosteroid prophylaxis requirements. Ongoing trials are evaluating these agents earlier in treatment and examining combinations awaiting approval in the near future [106].

Three CAR-T products (axi-cel, tisa-cel, and liso-cel) have also been approved for relapsed refractory follicular lymphoma and transformed FL. In a retrospective study, relapsed/refractory FL (*n* = 12) exhibited better OS following CAR-T treatment compared to transformed FL (*n* = 14) patients. The ORR was 100.0% vs. 92% with a 2-year PFS 75.0% vs. 66.7% and OS 100.0% vs. 73% for RR FL, higher compared to transformed FL (*p* = 0.04). A favorable remission state before CAR-T treatment correlated with improved PFS (*p*  =  0.009) [107].

In a pooled meta-analysis comparing CAR-T therapy to bispecific antibodies in relapsed/refractory FL, CAR-T demonstrated superior outcomes across all endpoints. Complete response rates were 82% vs. 64%, and overall response rates were 92% vs. 79%, favoring CAR-T. At one year, OS (95% vs. 77%) and PFS (75% vs. 56%) were also higher with CAR-T. These findings suggest that CAR-T offers more durable and robust responses, supporting its role as the preferred approach in this setting, while bispecific antibodies remain a clinically meaningful alternative [108].

CAR-T therapy may offer durable remission but is limited by prohibitive cost, poor accessibility, need for lymphodepletion, and notable toxicity, including cytokine release syndrome (CRS) and immune effector cell-associated neurotoxicity syndrome (ICANS), prolonged cytopenias, and infections [109,110,111]. Bispecific antibodies, by contrast, are readily available off the shelf, better tolerated, and more amenable to combination approaches, especially for older and frailer individuals who are not candidates for CAR-T [112].

Key unanswered questions remain around how best to sequence available therapies in the relapsed setting, including whether bispecific antibodies or CAR-T should be prioritized upfront, how to identify patients who would benefit from early aggressive immunotherapy, and whether deploying these agents in earlier lines of treatment will translate into improved long-term outcomes.

The amplification of programmed death ligands is rarely observed in FL tumor cells. Despite the transformative impact of checkpoint inhibitors across many malignancies, single agent use in FL has proven largely disappointing, with minimal response rates observed in clinical trials [113,114,115]. Combining checkpoint inhibitors with anti-CD20 therapy has shown more encouraging results. In a phase II trial, pembrolizumab plus rituximab in 30 patients with rituximab-sensitive relapsed FL yielded an ORR of 67%, CR rate of 50%, and median PFS of 13 months [116]. A phase Ib study of atezolizumab combined with obinutuzumab in 26 patients produced an ORR of 54%, CR rate of 23%, and median PFS of 9 months [117]. Triplet regimens have also been explored; atezolizumab, obinutuzumab, and lenalidomide achieved a CR rate of 72% and a 3-year PFS of 68% [118], while the addition of polatuzumab vedotin [119] to atezolizumab and obinutuzumab proved less effective and more toxic. These findings highlight the potential of combination approaches, though further investigation is needed to define optimal regimens and better understand the role of checkpoint inhibitors alongside other targeted agents.

## 5. Mantle Cell Lymphoma (MCL)

Mantle cell lymphoma (MCL) is a rare, incurable B-cell NHL characterized by CD5, BCL2, and cyclin D1 expression driven by t (11;14) translocation, presenting heterogeneously from indolent leukemic variants to aggressive nodal disease. Frontline management relies on rituximab-based chemoimmunotherapy [120]—with cytarabine-based induction and ASCT consolidation in eligible patients, or less intensive regimens otherwise—though BTK inhibitors are increasingly being incorporated into first-line strategies [121,122], prompting ongoing debate about the necessity of ASCT. Despite these advances, outcomes following BTKi failure remain dismal, with a median OS of only 2–10 months, underscoring a critical unmet need for more effective salvage therapies.

The t(11;14) translocation represents the defining genetic aberration of mantle cell lymphoma (MCL), although cryptic rearrangements involving immunoglobulin regulatory elements may serve as alternative oncogenic mechanisms in a subset of cases. This immunoglobulin enhancer hijacking mechanism occurs recurrently in cyclin D1-negative MCL cases that exhibit cyclin D2 or cyclin D3 overexpression. While MCL is generally classified among the most aggressive lymphomas, a minority of cases demonstrate an initially indolent clinical trajectory that may not require immediate therapeutic intervention. Non-nodal MCL (nnMCL) can be distinguished from conventional MCL (cMCL) by the presence of IGHV somatic hypermutations, leukemic presentation without lymphadenopathy, and a reduced burden of genomic alterations.

Although cyclin D1 dysregulation is considered the initiating oncogenic event [26], it is insufficient for complete malignant transformation of B-cell clones. Secondary somatic alterations affecting multiple cancer hallmark pathways are required for full lymphomagenesis, including cell cycle regulation (CDKN2A, CDK4, RB1) [123], DNA damage response (*TP53*, *ATM*, *CDKN2A*, *MYC*), epigenetic modification (*KMT2D*, *SMARCA4*, *NSD2* [124]), and *NF-κB* signaling (*BIRC3*, *NFKBIE*, *TNFAIP3*) [125]. Additionally, *SOX11* appears to collaborate with cyclin D1 in MCL pathogenesis by regulating complex transcriptional programs that enhance tumor aggressiveness [126]. The enhanced understanding of MCL pathogenesis through next-generation sequencing technologies, combined with integrated multidisciplinary approaches spanning molecular biology, pathology, and clinical research, has identified novel therapeutic targets and management strategies for aggressive MCL treatment.

The t(11;14)(q13;q32) translocation serves as the shared initiating event in MCL, but diverges into two biologically distinct pathogenic pathways. The more common conventional MCL (cMCL) arises from naïve-like pre-germinal center B cells, characterized by unmutated or minimally mutated IGHV, high genomic complexity, frequent SOX11 expression, and an aggressive clinical course [127]. The second subtype, leukemic non-nodal MCL (nnMCL), by contrast, arises from post-germinal center memory-like B cells and carries a distinct biological profile: mutated IGHV genes, absent SOX11 expression, and a relatively stable genome with low complexity [128,129,130]. Clinically, it follows an indolent course, owing in part to its limited angiogenic and tumor-invasive capacity. These divergent cellular origins are epigenetically preserved; both subtypes retain DNA methylation signatures that mirror their respective normal B-cell counterparts, naïve-like in cMCL and memory-like in nnMCL.

Despite sharing broadly similar global gene expression profiles, cMCL and nnMCL diverge considerably at the genetic and molecular levels. cMCL is defined by *SOX11* overexpression and genomic instability, with a tendency to accumulate progressive chromosomal aberrations over time. These biological features translate clinically into widespread lymphadenopathy at presentation and an aggressive disease trajectory [131,132]. nnMCL, in contrast, typically manifests as an indolent disease presenting predominantly as leukemic involvement [133,134] and is amenable to a watchful waiting approach without compromising outcomes [133]. Nevertheless, the disease can evolve over time, acquiring TP53 mutations, 17p deletions, and increasing genomic instability, which confer a poor prognosis [133]. To reliably distinguish between subtypes, a 16-gene (L-MCL16) assay on the NanoString platform was developed and validated in 70 MCL patients with leukemic presentation. Combined with genomic complexity and *TP53/CDKN2A* alterations, the assay demonstrated significant prognostic value, with nnMCL patients achieving a 3-year OS of 92% versus 69% in cMCL (*p* = 0.006) [134].

In MCL, brexucabtagene autoleucel (brexu-cel) was evaluated in the pivotal phase 2 ZUMA-2 trial [135]. Among sixty evaluable patients, the overall response rate (ORR) was 93% (56/60), with a complete response rate (CRR) of 67% (40/60). Notably, responses were robust across high-risk subgroups, including those refractory to prior BTK inhibitors (ORR 92%), TP53-mutated disease (ORR 100%), blastoid morphology (ORR 93%), and high Ki-67 (ORR 94%). At 3-year follow-up, the median (PFS), duration of response (DOR), and OS were 25.8, 28.2, and 46.6 months, respectively [136]. These findings led to FDA approval of brexu-cel in patients with relapsed MCL following BTK inhibitor therapy.

A second CAR-T-cell product, lisocabtagene maraleucel (liso-cel), has received FDA approval for R/R MCL following at least two prior lines of therapy, based on the results of the Transcend NHL 001 study [99]. Among seventy-four evaluable patients, the ORR was 86.5% (64/74) and the CRR was 74.3% (55/74), maintained across high-risk subgroups including blastoid (ORR 70.4%), TP53-mutated disease (ORR 89.5%), and high Ki-67 (ORR 85.5%). At a median follow-up of 22.8 months, the median DOR was 15.7 months and the median OS among patients achieving a CR was 36.3 months.

From a safety standpoint, liso-cel demonstrated a more favorable toxicity profile compared to brexu-cel. Serious treatment-emergent adverse events (TEAEs) occurred in 86% of patients, most commonly anemia, thrombocytopenia, and neutropenia. CRS occurred in 61% of patients, with only one grade ≥4 event, and grade 3–4 neurologic events occurred in just 9% of patients, a notably lower rate of ICANS than that observed with brexu-cel. Overall, liso-cel represents an effective and comparatively well-tolerated option.

Glofitamab is a novel BsAb with bivalent CD20 and monovalent CD3 binding. In a phase I/II trial, patients with R/R MCL who had received at least two prior lines of therapy were treated with fixed-duration glofitamab for twelve 21-day cycles [137]. To mitigate CRS risk, all patients received obinutuzumab pretreatment 7 days prior to the first glofitamab followed by step-up dosing (2.5 mg → 10 mg → 30 mg) to reach the target full dose. Among 60 evaluable patients, the ORR was 85% (51/60) and CRR was 78.3% (47/60), with a median DOR of 16.2 months and median duration of CR of 15.4 months [138]. Regarding safety, CRS was the most common adverse event, occurring in 70% of patients, with higher rates and severity observed in the obinutuzumab 1g cohort. ICANS occurred in only three patients (5%), with no grade ≥ 3 events. Overall, fixed-duration glofitamab demonstrated impressive and durable responses with a manageable toxicity profile in a heavily pretreated population.

Mosunetuzumab, another CD20 BITE antibody, was also initially evaluated as a single agent in a phase I/Ib study of R/R NHL, which included 13 patients with MCL [139]. Results were disappointing, with an ORR of 30.8% (4/13) and CRR of 23.1% (3/13), and approximately 45% of MCL patients experienced progressive disease. Given the limited sample size and modest response rates, single-agent mosunetuzumab did not appear to be a viable strategy in R/R MCL. In contrast, mosunetuzumab combined with polatuzumab vedotin (M-Pola) demonstrated more encouraging activity in a phase Ib/II study of 20 BTKi-pretreated R/R MCL patients [140]. The ORR was 75% (15/20) and CRR was 70% (14/20). CRS occurred in 50% of patients but was limited to grades 1–2, and three low-grade ICANS events were observed. Overall, M-Pola demonstrated favorable efficacy with a manageable toxicity profile, representing a potentially promising option in this high-risk population even after CAR-T failure.

## 6. Post-Transplant Lymphoproliferative Disorders (PTLDs)

EBV-positive PTLD presents a significant therapeutic challenge in transplant recipients, where immunologic strategies have become increasingly critical. While rituximab-based anti-CD20 immunotherapy remains the recognized first-line approach per AST, ECIL-6, and NCCN guidelines, it fails in up to 50% of cases following allo-HSCT, leaving no standardized second-line option. This unmet need has propelled adoptive cell immunotherapy to the forefront, and particularly EBV-specific CTLs, with third-party “off-the-shelf” HLA-matched products emerging as the most practical solution.

## 7. Future Directions in Immunotherapy of Hematological Malignancies

Future studies include moving CAR-T to earlier lines, as seen in frontline trials (ZUMA-23), as well as next-generation CAR-T strategies which include dual-targeting (CD19/CD20, CD19/CD22) [141], allogeneic “off-the-shelf” CAR-T [142], and armored CAR-T including fourth- [143] and fifth-generation [144] CAR-T constructs, as well as CAR-NK cells [145]. The frontline integration of BsAb is also being explored in NHL trials (EPCORE DLBCL-2, SKYGLO, OLYMPIA-3). Novel bispecific and trispecific antibodies include (CD79b × CD20 × CD3) [145].

Antibody–drug conjugates (ADCs) are used for initial cytoreduction as well as bridging prior to CAR-T therapy. ADCs work by targeted cytotoxic payload delivery. Polatuzumab vedotin is an ADC targeting CD79b which is used in Pola-R-CHP frontline and in Pola-BR in R/R. Loncastuximab tesirine is another attractive ADC that targets CD19, as shown in the LOTIS-5 update. Tafasitamab is a CD19 antibody, and lenalidomide is another option for DLBCL (L-MIND) and in combination with R2 for FL. Other emerging ADC targets include CD22 and CD37 ADCs, which are also explored in B-cell lymphoproliferative disorders.

Immune Checkpoint Inhibitors (ICIs): Combination strategies such as ICI + BsAb and ICI + chemotherapy are being explored to improve outcomes of both bispecific antibodies and CAR-T therapies. Other, newer emerging targets include *LAG-3*, *TIM-3*, and macrophage checkpoints (CD47, SIRPα), and these novel therapies are currently underway.

Adoptive Cell Therapy Beyond CAR-T: Future approaches include EBV-specific cytotoxic T lymphocytes (EBV-CTLs), e.g., tabelecleucel in PTLD and CAR-NK cells, innate immune harnessing, and CAR-macrophage approaches, as well as tumor-infiltrating lymphocyte (TIL) therapy.

## 8. Conclusions

Future studies include moving CAR-T to earlier lines and using next-generation CAR-T strategies, including dual-targeting vs. allogeneic “off-the-shelf” CAR-T, as well as CAR-NK-cell therapies. The frontline integration of novel options including BsAb as well as ADCs in recent years has improved the outcomes of both indolent and aggressive lymphomas. The key questions are how to consider the sequencing of CAR-T compared to BsAbs and how to mitigate the toxicities of these novel therapies with improved access and costs, so that these treatments can be universally available. Understanding the biology of lymphomas and molecular insights will provide key answers to these puzzling questions.

## Figures and Tables

**Table 1:** Novel therapies in lymphoma.

T-Cell-Engaging or -Derived Immunotherapies	
CAR-T-cell therapy	Axicabtagene ciloleucel, Tisagenlecleucel, Lisocabtagene maraleucel
Bispecific antibodies	Epcoritamab (CD20 × CD3), Glofitamab (CD20 × CD3), Mosunetuzumab, Odronextamab, TNB-486
Trispecific antibodies	CD19 × CD3 × CD2 formats (in development)
**NK-Cell- and Myeloid Cell-Engaging Therapies**	
Monoclonal antibodies	Tafasitamab (anti-CD19)
NK-cell therapy	Unmodified (allogeneic) NK cells, CAR-engineered NK cells
CD47/SIRPα axis blockade	Anti-CD47 or anti-SIRPα agents
CAR macrophages	CAR-M constructs targeting CD19/CD20
**Combination Therapies (Immunotherapy + Oncogenic Pathway Inhibitors)**	
B-cell surface marker combinations	CD19-directed: Tafasitamab, Loncastuximab tesirine; CD20-directed: Rituximab; CD79b-directed: Polatuzumab vedotin; CD20 × CD3: Glofitamab, Mosunetuzumab, Epcoritamab, Odronextamab
Immune checkpoint inhibition	Targets: *PD-1/PD-L1*, *TIGIT*, *LAG-3*, *TIM-3*
TME reprogramming/remodeling	Myeloid-targeting agents, T-cell-based approaches
Small molecules for combination	Lenalidomide, Ibrutinib

Abbreviations: CAR, chimeric antigen receptor; LAG-3, lymphocyte-activation gene 3; NK, natural killer; TIGIT, T-cell immunoreceptor with Ig and ITIM domains; TIM-3, T-cell immunoglobulin and mucin-domain containing-3; TME, tumor microenvironment.

**Table 2:** FDA-approved targeted therapies and immunotherapies in lymphoproliferative disorders (1997–2023).

No.	Approval Date	Drug/Regimen	Drug Class	Indication	Disease/Condition
**Drugs 1–13 of 26|November 1997–October 2017**					
1	November 1997	Rituximab	mAb (anti-CD20)	R/R low-grade lymphoma	B-cell Lymphoma
2	February 2006	Rituximab + CHOP	mAb + Chemotherapy	TN DLBCL	Diffuse Large B-cell Lymphoma
3	October 2009	Ofatumumab	mAb (anti-CD20)	R/R CLL	Chronic Lymphocytic Leukemia
4	August 2011	Brentuximab Vedotin	ADC (anti-CD30)	R/R HL or ALCL	Hodgkin Lymphoma/ALCL
5	June 2013	Lenalidomide	Immunomodulatory	R/R MCL	Mantle Cell Lymphoma
6	November 2013	Obinutuzumab + Chlorambucil	mAb (anti-CD20) + Chemo	TN CLL	Chronic Lymphocytic Leukemia
7	November 2013	Ibrutinib	BTK Inhibitor	R/R MCL	Mantle Cell Lymphoma
8	July 2014	Rituximab + Idelalisib	mAb + PI3K Inhibitor	R/R CLL	Chronic Lymphocytic Leukemia
9	April 2016	Venetoclax	BCL-2 Inhibitor	R/R CLL	Chronic Lymphocytic Leukemia
10	May 2016	Nivolumab	PD-1 Inhibitor (mAb)	R/R HL	Hodgkin Lymphoma
11	March 2017	Pembrolizumab	PD-1 Inhibitor (mAb)	R/R HL	Hodgkin Lymphoma
12	October 2017	Acalabrutinib	BTK Inhibitor	R/R MCL	Mantle Cell Lymphoma
13	October 2017	Axi-cel (Axicabtagene Ciloleucel)	CAR-T-cell Therapy	R/R NHL	Non-Hodgkin Lymphoma
**Drugs 14–26 of 26|May 2018–June 2023**					
14	May 2018	Tisa-cel (Tisagenlecleucel)	CAR-T-cell Therapy	R/R LBCL	Large B-cell Lymphoma
15	June 2018	Rituximab + Venetoclax	mAb + BCL-2 Inhibitor	R/R CLL	Chronic Lymphocytic Leukemia
16	August 2018	Mogamulizumab	mAb (anti-CCR4)	MF or SS	Mycosis Fungoides/Sezary Syndrome
17	August 2018	Rituximab + Ibrutinib	mAb + BTK Inhibitor	R/R WM	Waldenstrom Macroglobulinemia
18	January 2019	Obinutuzumab + Ibrutinib	mAb + BTK Inhibitor	TN CLL	Chronic Lymphocytic Leukemia
19	May 2019	Rituximab + Lenalidomide	mAb + Immunomodulatory	R/R FL or MZL	Follicular/Marginal Zone Lymphoma
20	November 2019	Obinutuzumab + Acalabrutinib	mAb + BTK Inhibitor	CLL	Chronic Lymphocytic Leukemia
21	November 2019	Zanubrutinib	BTK Inhibitor	R/R MCL	Mantle Cell Lymphoma
22	July 2020	Brexu-cel (Brexucabtagene Autoleucel)	CAR-T-cell Therapy	R/R MCL	Mantle Cell Lymphoma
23	February 2021	Liso-cel (Lisocabtagene Maraleucel)	CAR-T-cell Therapy	R/R LBCL	Large B-cell Lymphoma
24	January 2023	Pirtobrutinib	BTK Inhibitor (non-covalent)	R/R MCL	Mantle Cell Lymphoma
25	May 2023	Epcoritamab	Bispecific Ab (CD3xCD20)	R/R DLBCL	Diffuse Large B-cell Lymphoma
26	June 2023	Glofitamab	Bispecific Ab (CD3xCD20)	R/R DLBCL	Diffuse Large B-cell Lymphoma

Abbreviations: ADC: antibody–drug conjugate; BTK: Bruton’s tyrosine kinase; CAR-T: chimeric antigen receptor T cell; CLL: chronic lymphocytic leukemia; DLBCL: diffuse large B-cell lymphoma; FL: follicular lymphoma; HL: Hodgkin lymphoma; LBCL: large B-cell lymphoma; mAb: monoclonal antibody; MCL: mantle cell lymphoma; MF: mycosis fungoides; MZL: marginal zone lymphoma; NHL: non-Hodgkin lymphoma; R/R: relapsed/refractory; SS: Sezary syndrome; TN: treatment-naïve; WM: Waldenstrom macroglobulinemia.

**Table 3:** Genetic mutations and pathway dysregulation by hematological malignancy.

Disease	Genetic Alteration/Chromosomal Change	Pathway/Mechanism Affected	Clinical Relevance
CML	t(9;22) → *BCR-ABL1* fusion (Philadelphia chromosome)	Persistent tyrosine kinase activity → uncontrolled proliferation	Defining marker; imatinib/TKI target
B-ALL	t(9;22) → *BCR-ABL1* fusion	Aberrant kinase signaling	Poor-risk subset; TKI-responsive
MCL	t(11;14)(q13;q32) → *CCND1* overexpression	Cell cycle dysregulation (G1/S checkpoint)	Defining translocation; malignant transformation
MCL	*TP53* mutation/del(17p)	DNA damage response failure	Chemo & BTK inhibitor resistance; poor prognosis
Follicular Lymphoma	*BCL-2* upregulation via t(14;18)	Apoptosis resistance	Malignant cell survival; venetoclax target
Follicular Lymphoma	*KMT2D*, CREBBP, *EZH2* loss-of-function	Histone modification; germinal center dysregulation	Lymphomagenesis; immunotherapy resistance
DLBCL	*EZH2* gain-of-function (~20% of cases)	↑ H3K27me3 → suppressed differentiation, immune evasion	Tazemetostat target; immunotherapy resistance
DLBCL	*CREBBP* loss-of-function mutations	MHC class II transcriptional silencing	Resistance to immunotherapy
AML	*FLT3-ITD/FLT3-TKD* mutations (~25–33%)	STAT5, PI3K, MAPK activation	Adverse prognosis; midostaurin/gilteritinib target
AML	*NPM1*, *IDH1/2*, *DNMT3A*, *TET2*, *TP53* mutations	Epigenetic reprogramming; aberrant transcription	Define subgroups; guide risk stratification & therapy
AML/MDS	*IDH1/IDH2* mutations	Widespread CpG methylation gains	IDH inhibitor target (enasidenib, ivosidenib)
CLL	*TP53* mutation/del(17p)	DNA damage response; apoptosis failure	Highest adverse prognosis; BTK/BCL-2 inhibitor indication
CLL	*NOTCH1*, *SF3B1*, *ATM*, *BIRC3* mutations	BCR signaling, NF-κB, RNA splicing	Unmutated IGHV; shorter TTT, worse PFS/OS
CLL	*BTK C481S/PLCG2* mutations	Non-covalent BTK binding; BCR signaling escape	Ibrutinib resistance; non-covalent BTK inhibitor rationale
CLL/MCL	Chronic active *BCR* signaling	*SYK → PI3K → BTK → NF-* *κ* *B/MAPK/AKT-mTOR*	Validated target; BTK inhibitor-sensitive
PV	*JAK2V617F* mutation (>95%)	Constitutive JAK-STAT activation	Proliferative phenotype; ruxolitinib target
ET/MF	*JAK2V617F* mutation	JAK-STAT pathway activation	Myeloproliferative phenotype; JAK inhibitor-responsive

Abbreviations: AML, acute myeloid leukemia; B-ALL, B-cell acute lymphoblastic leukemia; BCR, B-cell receptor; BCR-ABL1, breakpoint cluster region–I Abelson murine leukemia 1; *BIRC3*, baculoviral IAP repeat containing 3; *BTK*, Bruton’s tyrosine kinase; CCND1, cyclin D1; CLL, chronic lymphocytic leukemia; CML, chronic myeloid leukemia; *CREBBP*, CREB binding protein; del(17p), deletion of chromosome 17p; DLBCL, diffuse large B-cell lymphoma; *DNMT3A*, DNA methyltransferase 3 alpha; ET, essential thrombocythemia; EZH2, enhancer of zeste homolog 2; FLT3-ITD, FMS-like tyrosine kinase 3 internal tandem duplication; *FLT3-TKD*, FMS-like tyrosine kinase 3 tyrosine kinase domain; *IDH1/2*, isocitrate dehydrogenase 1/2; *IGHV*, immunoglobulin heavy-chain variable region; *JAK2*, Janus kinase 2; *JAK-STAT*, Janus kinase–signal transducer and activator of transcription; *KMT2D*, lysine methyltransferase 2D; MAPK, mitogen-activated protein kinase; MCL, mantle cell lymphoma; MDS, myelodysplastic syndrome; MF, myelofibrosis; MHC, major histocompatibility complex; mTOR, mammalian target of rapamycin; NF-κB, nuclear factor kappa B; *NOTCH1*, notch homolog 1; *NPM1*, nucleophosmin 1; OS, overall survival; PFS, progression-free survival; PI3K, phosphatidylinositol 3-kinase; PLCG2, phospholipase C gamma 2; PV, polycythemia vera; SF3B1, splicing factor 3b subunit 1; SYK, spleen-associated tyrosine kinase; *TET2*, tet methylcytosine dioxygenase 2; TKI, tyrosine kinase inhibitor; *TP53*, tumor protein 53; TTT, time to first treatment.

## Data Availability

No new data were created or analyzed in this study. Data sharing is not applicable to this article.

## Abbreviations

ADC: Antibody–Drug Conjugate; BTK: Bruton Tyrosine Kinase; CAR-T: Chimeric Antigen Receptor T Cell; CLL: Chronic Lymphocytic Leukemia; DLBCL: Diffuse Large B-cell Lymphoma; FL: Follicular Lymphoma; HL: Hodgkin Lymphoma; LBCL: Large B-cell Lymphoma; mAb: Monoclonal Antibody; MCL: Mantle Cell Lymphoma; MF: Mycosis Fungoides; MZL: Marginal Zone Lymphoma; NHL: Non-Hodgkin Lymphoma; R/R: Relapsed/Refractory; SS: Sezary Syndrome; TN: Treatment-Naïve; WM: Waldenstrom Macroglobulinemia.
